# Evidence of neuroplasticity with brain–computer interface in a randomized trial for post-stroke rehabilitation: a graph-theoretic study of subnetwork analysis

**DOI:** 10.3389/fneur.2023.1135466

**Published:** 2023-06-06

**Authors:** Zhen-Zhen Ma, Jia-Jia Wu, Xu-Yun Hua, Mou-Xiong Zheng, Xiang-Xin Xing, Jie Ma, Chun-Lei Shan, Jian-Guang Xu

**Affiliations:** ^1^Department of Rehabilitation Medicine, Longhua Hospital, Shanghai University of Traditional Chinese Medicine, Shanghai, China; ^2^School of Rehabilitation Science, Shanghai University of Traditional Chinese Medicine, Shanghai, China; ^3^Engineering Research Center of Traditional Chinese Medicine Intelligent RehabilitationMinistry of Education, Shanghai, China; ^4^Department of Rehabilitation Medicine, Yueyang Hospital of Integrated Traditional Chinese and Western Medicine, Shanghai University of Traditional Chinese Medicine, Shanghai, China; ^5^Department of Trauma and Orthopedics, Yueyang Hospital of Integrated Traditional Chinese and Western Medicine, Shanghai University of Traditional Chinese Medicine, Shanghai, China

**Keywords:** brain–computer interface, motor imagery, graph-theoretic analysis, stroke rehabilitation, brain plasticity

## Abstract

**Background:**

Brain–computer interface (BCI) has been widely used for functional recovery after stroke. Understanding the brain mechanisms following BCI intervention to optimize BCI strategies is crucial for the benefit of stroke patients.

**Methods:**

Forty-six patients with upper limb motor dysfunction after stroke were recruited and randomly divided into the control group or the BCI group. The primary outcome was measured by the assessment of Fugl–Meyer Assessment of Upper Extremity (FMA-UE). Meanwhile, we performed resting-state functional magnetic resonance imaging (rs-fMRI) in all patients, followed by independent component analysis (ICA) to identify functionally connected brain networks. Finally, we assessed the topological efficiency of both groups using graph-theoretic analysis in these brain subnetworks.

**Results:**

The FMA-UE score of the BCI group was significantly higher than that of the control group after treatment (*p* = 0.035). From the network topology analysis, we first identified seven subnetworks from the rs-fMRI data. In the following analysis of subnetwork properties, small-world properties including *γ* (*p* = 0.035) and *σ* (*p* = 0.031) within the visual network (VN) decreased in the BCI group. For the analysis of the dorsal attention network (DAN), significant differences were found in assortativity (*p* = 0.045) between the groups. Additionally, the improvement in FMA-UE was positively correlated with the assortativity of the dorsal attention network (*R* = 0.498, *p* = 0.011).

**Conclusion:**

Brain–computer interface can promote the recovery of upper limbs after stroke by regulating VN and DAN. The correlation trend of weak intensity proves that functional recovery in stroke patients is likely to be related to the brain’s visuospatial processing ability, which can be used to optimize BCI strategies.

**Clinical Trial Registration:**

The trial is registered in the Chinese Clinical Trial Registry, number ChiCTR2000034848. Registered 21 July 2020.

## 1. Introduction

Stroke is ranked as the second leading cause of death and the third most common cause of disability worldwide (1). Deficiencies that include loss of motor function, cognition, speech, or mood regulation lead to a severe burden after stroke and result in poor quality of life. Especially, recovery from upper extremity dysfunction is often inadequate and unsatisfactory from exercise rehabilitation, due to the severity of the injury and the limited time for treatment focused on upper extremity recovery (2). Therefore, there is an urgent need for innovative tools that can facilitate the successful recovery of motor function. Brain–computer interfaces (BCIs) represent a promising rehabilitation strategy, which can control external devices by modulating their sensorimotor rhythms (SMRs) generated by neuronal units of the sensorimotor gyrus. During motor attempts or imagery, the amplitude of the SMR declined, a modulation called event-related desynchronization (ERD), which can be translated into control commands from external devices. There was no actual physical movement necessary for controlling BCI-based devices, even stroke survivors can modulate or manipulate with severe chronic motor deficits. The generated control commands during motor attempts or imagery are independent of residual motor function (3), providing favorable conditions for the rehabilitation of patients with moderate-to-severe disabilities in the upper limbs.

Motor imagery (MI, the mental representation of action without actual movement) is a therapy-relevant technique that promotes motor recovery after neurological disorders (4, 5). MI shares psychological and neural foundations with physical exercises (6, 7). Neurophysiological recordings yielded specific changes in cerebral activations during MI, resembling movements actually performed and affecting neural representations of movements (8, 9). Numerous studies have shown that neural processes associated with motor imagery are attributed to the activation of the premotor and parietal areas, primary sensory-motor cortex, and subcortical regions such as basal ganglia and the cerebellum, as well as corticospinal pathways (9, 10). Therefore, MI has been widely used in BCI systems for neurorehabilitation applications, ranging from individuals with motor disabilities, severe muscular disorders, and paralysis to the restoration of limb movements. Due to the bidirectional interaction between the brain and the computer, MI-BCI systems are used to alter the brain functions of stroke patients through reorganizational processes.

Research has shown that BCI systems can help stroke patients (11–13). Such systems can induce the input–output properties of spinal cord circuits in real time, which can promote integrated neuroplasticity of affected corticospinal connections, effectively closing the loop to efferent brain signals coupled to afferent inputs (14, 15) and facilitate voluntary motor control (16). As demonstrated using BCIs to increase motor evoked potentials (MEPs) in stroke survivors, neural components are activated associatively, thus strengthening intra-cortical synaptic connections (17, 18). Furthermore, the real-time visualization of neurofeedback BCI embodied improves the ability of the disabled brain area, so that more participation of the ipsilateral hemisphere was engaged and motor function was improved compared to the random feedback (19). In addition to altering neuroplasticity by affecting neurophysiological parameters, BCI applications have also been shown to induce functional improvements (20–23).

However, fMRI brain mechanisms involved in BCI remain sparse. fMRI analysis suggested that an integrated BCI-guided robotic hand training intervention may contribute to neuroplasticity in stroke patients, with interhemispheric asymmetry significantly associated with training effects and the integrity of the M1-M1 anatomical connection (24). Li et al. found that the motor performance and the cortical motor induction of cortical MEPs had improved significantly, but the fractional anisotropy (FA) value of the lesion area was not significantly improved after 4 weeks of treatment with a brain–computer interface-operated lower limb rehabilitation robot (17). Although BCI-based approaches appear promising for limb rehabilitation, sufficient evidence for widely used clinical application is still lacking and more debate on its research is needed.

Substantial systematic reviews have examined the effects of BCIs on limb motor rehabilitation after stroke (25, 26). It is generally accepted that there is considerable heterogeneity in the models and implementation methods (especially the duration of treatment) of BCIs in clinical applications (27) and that the effects of BCI training vary from person to person (28). The lack of a control group (28) and random assignment in clinical studies prevented us from performing a valid analysis. Furthermore, a limited number of studies do not support the long-term follow-up effects of BCI training (29, 30). In addition, how BCI promotes patients’ motor function and what breakthroughs can be made in its further optimization still need deep research and demonstration. The study of mathematical models of complex networks, such as graph theory, provides excellent tools for understanding the organizational characteristics of brain networks after the BCI intervention. In the present study, we explored the effect of MI-based BCI on functional recovery and analyzed the network metric of functional networks by constructing functional brain networks to represent changes at the network level. A better understanding of the mechanism of application-induced neuroplasticity based on BCI can help to optimize the standardized application protocol of brain–computer interface with multiple feedback, making it a more practical and effective method for the treatment of upper extremity hemiplegia in the future.

## 2. Materials and methods

A total of 187 patients were screened in the outpatient clinic and inpatient of the Department of Neurology, Rehabilitation Medicine, and Acupuncture in a tertiary healthcare hospital in Shanghai. Stroke diagnosis was established clinically in all the patients. The study was approved by the local ethics committee, and the study protocol was registered in the Chinese Clinical Trial Registry (ChiCTR2000034848). All participants signed informed consent forms before enrollment according to the Helsinki Declaration.

### 2.1. Study design

A single-center, equal randomization (1:1 for two groups), single-blind, controlled, parallel-group study was designed which included pre- and post-intervention. Once enrolled, patients were randomly assigned to either control or BCI groups. The random allocation schedule was computer-generated through a simple randomization method that generates a random allocation sequence. Patients were instructed to enter the appropriate groups sequentially in a predefined sequence. After randomization, visits were scheduled pre- and post-intervention. Randomization, assessments, and data analysis were performed by a different individual not involved in the intervention.

### 2.2. Patients enrollment

**Figure 1:**
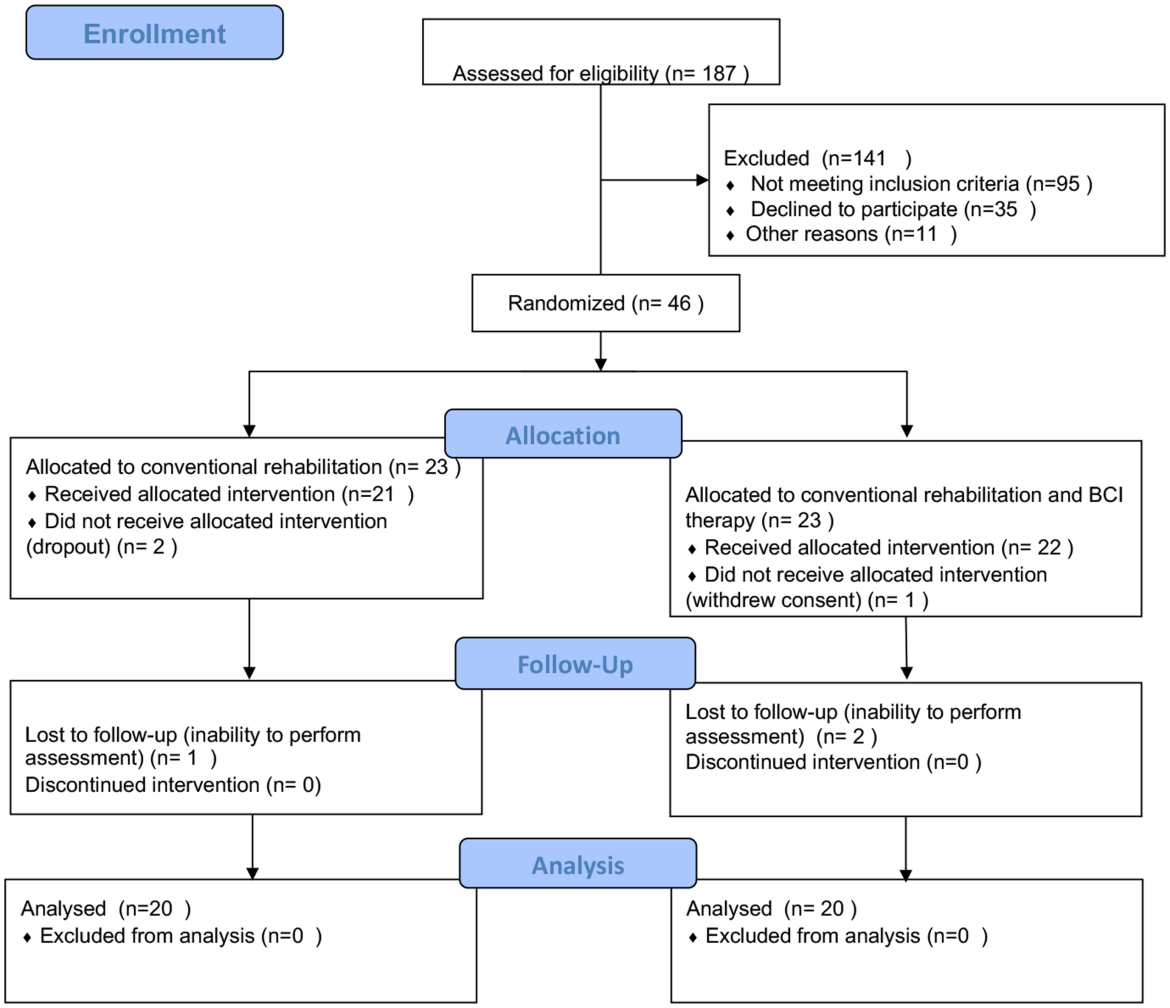
Protocol of the intervention.

Participants were selected based on the following inclusion criteria: age between 30 and 75 years old; duration of hemorrhagic or ischemic stroke ≥1 month and ≤ 12 months; a significant decrease in unilateral upper extremity motor function; clear consciousness, NIHSS (National Institute of Health stroke scale) ≥ 6 points (moderate stroke severity and above); no cognitive impairment, with the ability to understand instructions (score above 22 on the mini-mental state examination (MMSE)); agree to participate in this study and sign informed consent. An abbreviated list of exclusion criteria includes the following: other diseases or factors affecting limb movement; post-stroke patients with complex regional pain syndrome with significant pain and swelling; contraindications to an investigation by magnetic resonance; complicated with serious heart, liver, kidney, and blood system diseases, or infectious diseases; have participated in other studies or are participating in other clinical studies 6 months before enrollment (see Figure 1).

### 2.3. Intervention protocol

The two groups received conventional physical therapy and occupational therapy, including limb dominance exercise, muscle tension, and limb control training to improve functionality, balance, and daily activities, 5 times a week, for 10 times. In addition, the BCI group underwent additional BCI therapy for 40 min of individual sessions per day (5 days a week for 2 weeks) using the MI-based BCI system. Both groups continued to receive an appropriate clinical standard practice of medication, including aspirin or other antiplatelet or antithrombotic drugs, serum lipid-lowering agents, antihypertensive drugs, and hypoglycemic agents throughout the study as prescribed by the neurologist. All therapy sessions were delivered to patients by trained and experienced physiotherapists. One occupational therapist performing the assessments and data analysis was blinded to the intervention.

### 2.4. MI-based BCI system

**Figure 2:**
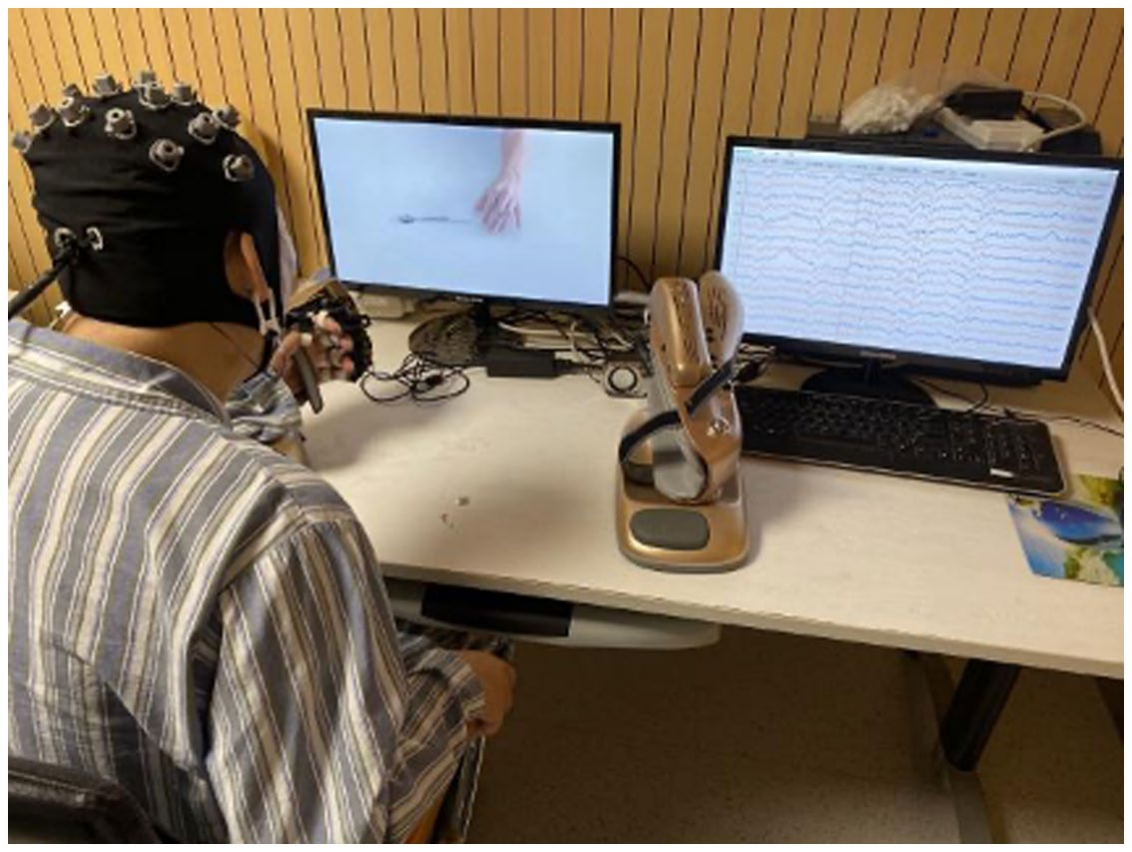
MI-based BCI training system and experimental setup. The Figureure panel presents the experimental setup with a patient sitting in front of a computer screen wearing an EEG cap. The patient performs the motor imagery of the corresponding activities that appear on the screen, such as reaching out to grab or place a spoon, a bowl, and a ball. Real-time online EEG signals can be presented on the right display and are decoded to control the neurostimulator of the robotic assistive arm. Robot-assisted arm training improves upper limb and arm function through passive movements of the person’s arm. The device displays experimental instructions through visual and auditory cues, and the patient simultaneously performs motor imagery according to the instructions, and then, the device provides feedback. Encouragement pictures such as “You’re amazing!” and “Just a little bit, keep working hard” will appear on the screen, and the small speaker will send out corresponding verbal rewards. At the same time, the robotic arm will help the disabled arm to perform passive flexion and extension activities. The device increases or decreases the difficulty of the task based on the patient’s participation in the task. Patients who are exposed to the equipment for the first time need to be trained so that they are familiar with and can participate in the entire experiment.

A set of brain–computer interface-assisted upper limb rehabilitation training systems based on motor imagery was used in this study. The system uses human–computer interaction to help patients participate in different activities of the upper limb for rehabilitation training. A 16-channel EEG acquisition device was used to collect EEG signals in the sensorimotor areas of the brain. The BCI performs online real-time processing of EEG signals for a single-trained online classification of μ-wave probabilistic models for imagining left-hand and right-hand movements. This method performs temporary Morlet wavelet filtering on the source signal adjusted by the subject, selects the appropriate time–frequency signal, and then classifies and processes the EEG signal according to the time–frequency algorithm. The signal acquisition is the characteristic motion signal of the amplitude-modulated μ rhythm in the range of 8 Hz–12 Hz measured at C3 and C4 and used to estimate its probability model. The error rate of this algorithm is not higher than 10%. Through the analysis of the real-time online characteristic signals of the electroencephalography (EEG) signal, the external devices are controlled to carry out corresponding activities. The device for the rehabilitation of the hand is RHB-II-L/RHB-II-R (registration certificate number: Yueji Note 20,182,190,967; see Figure 2).

During the task, the patient is placed in a dedicated chair in front of a personal computer (PC) monitor and left 40 cm away, with the paralyzed hand immobilized in the exoskeleton, while the intact hand rests flat on a table. Lessons on a stable platform with biofeedback take the form of game tasks based on using visual and auditory channels to support responses. On a monitor screen, the patient was given mental commands: to relax and to imagine the state of the muscles as the right or left-hand stretches or contracts. The patient imagines the functional activities of the paralyzed hand through the task prompts on the screen and the voice prompts synchronized with the speakers. Corresponding results emerged after identifying the classifier correctly performing the mental task, where only the paralyzed hand is involved in arm activity. Each working program of the task consisted of 3- to 8-min sessions with a 1- to 3-min break between each session depending on the tolerance of the patient; the number of performed programs was 3–4. Each movement process has a maximum of three chances. The task will prompt you to be awesome and give a passing grade or above; meanwhile, the exoskeleton extended or flexed its fingers. If the task fails to complete, a reminder that it is still a little bit close, please continue to work hard will be received. The duration of such training is determined individually in each case according to the patient’s abilities and subjective tolerance and is on average 40 min. The whole process of treatment is accompanied and guided by one professional doctor.

Usually, in the formal treatment stage, patients will receive 2–3 times of training to familiarize themselves with the experimental procedures. Patients are instructed to perform the imagery tasks based on a computer screen and voice prompts and to avoid blinking, coughing, chewing, and head and body movements (31).

### 2.5. Data collection

To detect an improvement in FMA-UE, which is in agreement with the study of Wang et al. (32) with a two-sided 5% significance level and a power of 90%, a sample size of 23 patients per group was necessary, given an anticipated dropout rate of 10%. The study started in March 2020 and stopped in January 2021. In total, 23 participants were allocated to the control group (three dropped out), and 23 were allocated to the BCI group (three dropped out). All patients went through motor function, related neuropsychological assessment, and BOLD function assessment pre- and post-intervention. Each one of the assessment times had an approximate duration of 120 min.

The upper extremity motor performance of stroke patients was evaluated using the Fugl–Meyer Assessment of Upper Extremity (FMA-UE) test pre- and post-intervention. The FMA-UE was our primary outcome measure in this study, which is a test based on the concept of sequential stages of motor return (33), including reflexes, the synergy of the upper extremities, and hand function. Each item is scored on an ordinal 3-point scale to represent a maximum motor score for the affected side, with a total score ranging from 0 (standing for hemiplegia) to 66 (standing for normal) (34). We also assessed clinical characteristics of patients at baseline, including FMA-LE (Fugl–Meyer Assessment of Lower Extremity), FMA (Fugl–Meyer Assessment), MMSE (mini-mental state examination), MBI (Modified Barthel Index), and the motor imagery ability assessed by the Kinesthetic and Visual Imagery Questionnaire (KVIQ) (35). The clinical characteristics of the patients studied are described in Table 1.

**Table 1:** Comparison of demographic data and clinical characteristics.

		Control group (*n* = 20)	BCI group (*n* = 20)	Statistics (*t*/*Z*)	*p*-value
*Generalized characteristics*					
Age		58.30 ± 11.23	50.90 ± 12.64	1.479	0.058
Time since stroke (months)		6.45 ± 3.38	5.90 ± 2.99	0.841	0.589
Gender	Male	15	16	0	1
	Female	5	4		
Etiology	Ischemic	19	14	2.771	0.096
	Hemorrhagic	1	6		
Paralysis side	Left	9	9	0	1
	Right	11	11		
Clinical Characteristics					
MMSE		26.20 ± 3.50	26.20 ± 3.09	0	1
FMA-UE		20.75 ± 10.77	20.80 ± 11.21	−0.014	0.989
FMA-LE		21.25 ± 6.58	18.55 ± 8.06	1.161	0.253
FMA		55.25 ± 20.59	47.35 ± 17.26	1.314	0.196
MBI		73.50 ± 23.83	59.60 ± 22.97	1.692	0.099
VIQ		66.85 ± 11.52	66.10 ± 12.42	0.198	0.844
KIQ		59.00 ± 15.68	59.25 ± 12.96	−0.055	0.956

MMSE, mini-mental state examination; FMA-UE, Fugl–Meyer Assessment of Upper Extremity; FMA-LE, Fugl–Meyer Assessment of Lower Extremity; FMA, Fugl–Meyer Assessment; MBI, modified Barthel Index; Kinesthetic and Visual Imagery Questionnaire (KVIQ); including Kinesthetic Imagery Questionnaire (KIQ), and Visual Imagery Questionnaire (VIQ).

*p < 0.05.

### 2.6. MRI acquisition and preprocessing

All participants underwent a whole-brain scan of resting-state fMRI (rs-fMRI) with a 3.0 Tesla scanner (SIEMENS AG, MAGNETOM Verio) using an 8-channel head coil. The complete fMRI scan lasts about 10 min, and patients were warned to close their eyes and remain still. All subjects suppressed the unexpected movement, and all were compliant during the fMRI scan. In the resting-state session, the following parameters were listed: interleaved scanning order, slice number = 43, repetition time (TR) = 3,000 ms, field of view (FOV) = 240 × 240 mm^2^, flip angle (FA) = 90 degrees, interslice space = 3 mm, with no interval, number of acquisitions = 200.

The preprocessing and analysis of fMRI data were performed using SPM12 (Wellcome Trust Centre for Neuroimaging, London; https://www.fil.ion.ucl.ac.uk/spm/software/spm12/) on the MATLAB 2014a platform, and the graph-theoretical network analysis was developed on the GRaph thEoreTical Network Analysis (GRETNA) (http://www.nitrc.org/projects/gretna/) toolbox (36). To ensure consistency on both sides of the patient and the establishment of standardized parameters, the brain images of patients with right-sided lesions were flipped to the midsagittal plane so that the affected hemispheres of all patients corresponded to the left-sided brain.

After discarding the first 10 volumes of each fMRI run, slice timing was performed to correct for inconsistencies in time collection in the preprocessing step. The point-to-point head motion and mean head motion were then estimated for the subjects to control for the motion-induced artifacts. Next, the data were normalized to the stereotactic Montreal Neurological Institute (MNI) space using the T1 SPM template and resulted in voxels of 3 × 3 × 3 mm^3^. Normalized images were smoothed with a 6 mm full width at a half-maximum isotropic Gaussian kernel.

### 2.7. Node and edge definition

The topological analysis of functional subnetworks was performed using GIFT software (Group ICA of fMRI Toolbox, version 4.0, http://icatb.sourceforge.net). We constructed subnetworks by decomposing preprocessed rs-fMRI data into independent components (ICs). Generally, group ICA for such multi-subject analysis uses a concatenated approach coupled with back reconstruction (37). The images were first dimensionally reduced using principal component analysis (PCA) and then temporally concatenated and reduced using an expectation–maximization algorithm at the group level to extract 40 spatial components. Furthermore, the infomax ICA algorithm was performed in ICASSO for 100 repetitions to verify its robustness (38).

After estimating the aggregated spatial maps, subject-specific spatial patterns and temporal courses were extracted through the back-reconstruction approach. Spatial weight maps and the temporal course were generated for all subjects, which revealed the likelihood that a voxel belongs to one particular component. Then, we thresholded these maps at the group level after a Z-transformation of the spatial weight map.

**Figure 3:**
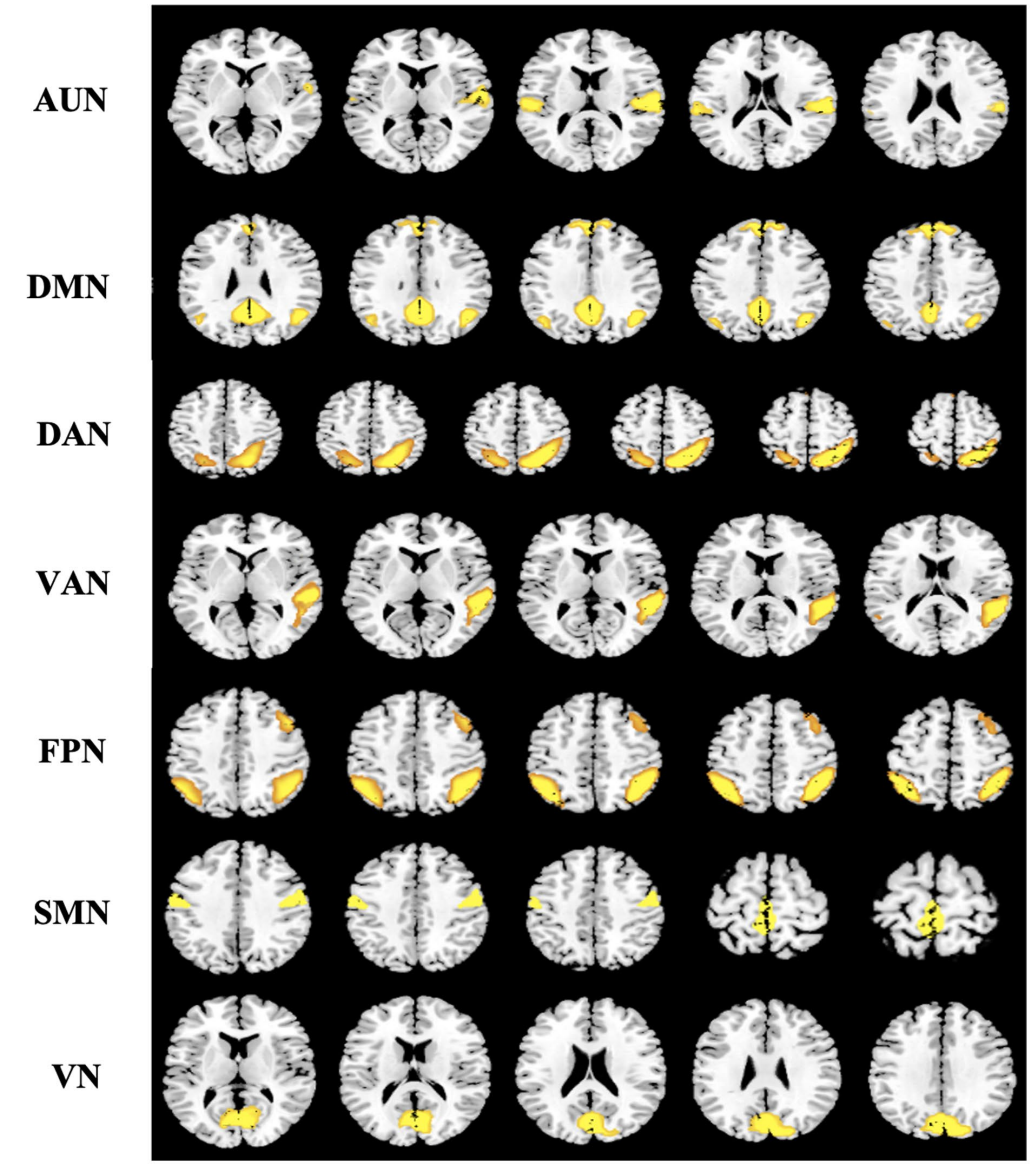
Spatial distribution pattern of seven potential subnetworks extracted from resting-data by ICA. Seven components resemble the RSNs described in a previous study (39) and consist of regions known to be involved in the auditory network (AUN), default mode network (DMN), dorsal attention network (DAN), ventral attention network (VAN), frontoparietal network (FPN), sensorimotor network (SMN), and visual network (VN). Images (axial views) are *t*-statistics overlayed on the average high-resolution scan transformed into MNI152. ICA, independent component analysis; RSNs, resting-state networks; MNI, Montreal Neurological Institute.

We selected meaningful ICs from resting-state networks (RSNs) described in previous studies by spatial ordering and visual inspection (39). Seven ICs of interest were identified in this study: the auditory network (AUN), default mode network (DMN), dorsal attention network (DAN), ventral attention network (VAN), frontoparietal network (FPN), sensorimotor network (SMN), and visual network (VN; Figure 3) (40). For these subnetworks, we designated each voxel as the node and the voxel-voxel functional connectivity as an edge. In addition, we resampled the data and resized the voxel size to 2x or 3x smaller to reduce the large scale of the connectivity matrix, which significantly reduced the computational effort.

Then, we computed the network metrics of the subnetworks using a pre-selected sparse value (the ratio of the actual number of edges divided by the maximum possible number of edges in the network) to ensure the relative network organization after the functional connectivity matrices were obtained. In particular, the topological organization of networks was analyzed over a wide range of network sparsity (0.05–0.4) (41), where the small-world metrics were also analyzed (42). Network density was applied to each adjacent matrix in increments of 0.01 to reduce the computational dimension.

### 2.8. Global properties of subnetworks (secondary outcomes)

Graph theory analysis is a suitable and appreciable method for characterizing the topological properties of brain networks (43, 44). Commonly used network metrics, including small-world properties (clustering coefficient *C_p_*, characteristic path length *L_p_*, normalized clustering coefficient *γ*, normalized characteristic path length *λ,* and small-worldness *σ*), network efficiency properties (local efficiency *E_local_* and global efficiency *E_global_*), assortativity *r*, hierarchy *β,* and synchronization *S,* were calculated in this study. All network metrics were performed with GRETNA (36, 45).

The small-world parameters take into account modularized/specialized and distributed/ integrated information processing, greatly improving the efficiency of information transmission at a low cost. Therefore, small-world networks have a shorter *L_p_* than regular networks (high *C_p_* and long *L_p_*) but have a larger *E_local_* than random networks (low *C_p_* and short *L_p_*). To characterize the small-world property of the target network, we computed its corresponding values of *C_p_* and *L_p_* from the average of 100 random networks with equal node size and degree distribution (46). Compared with random networks, small-world networks have relatively high normalized clustering coefficients *γ* (*C_p_*/*C*_*p*rand_) >1 and a relatively lower normalized characteristic path length *λ* (*L_p_* / *L*_*p*rand_) ≈ 1 (47).

Global efficiency measures the efficiency of information transfer at the network level, which is the reciprocal of the harmonic mean of the minimum path length (48). While the local efficiency of a network measures fault tolerance in the network, it shows how efficiently communications can be exchanged when a given node’s first neighbor is eliminated (48).

Additionally, we evaluated the hierarchical nature of networks using the *β* parameter (49), which defines the magnitude of the power–law relationship between the clustering coefficient (*C_p_*) and degree (k): $\mathrm{Cp}\approx k^{-\beta }$ (50). In a network with hierarchy organizations, some highly correlated related nodes form a densely connected cluster. These generated clusters act as elements at the next level of the network and merge into a larger-scale interconnected cluster (51). We calculated the parameter *β* for the network using the log (C) versus log (k) plot fitted to the regression line.

Furthermore, assortativity reflects the tendency of nodes to associate these nodes with a similar number of edges, and it measures the correlation between the degree of a node and the average degree of its neighbors (52). A positive correlation means that closely connected nodes are more likely to be associated with other nodes of the same degree. Synchronization, the ratio of the next-smallest eigenvalue to the largest eigenvalue of the network coupling matrix, measures the likelihood of fluctuations occurring at all nodes in the same wave pattern.

### 2.9. Statistical analyses

The statistical analyses were conducted using SPSS software (version 22; SPSS Inc., Chicago, IL), and the significance level was set at a *p*-value of < 0.05. Demographic and neurological characteristics were compared between the groups using the independent-sample *t*-test for continuous variables and *χ*^2^ test or Fisher’s exact test for categorical variables. Comparison of the primary outcome of FMA-UE before and after treatment in both groups was analyzed by repeated measures analysis of variance (ANOVA) and Tukey’s honest significant difference *post-hoc* tests where applicable, at a significance level of the *p*-value of < 0.05.

The area under the curve (AUC) over the sparsity range was used to conduct group comparisons of the metric. Statistical tests of topological measures between the groups were performed using an independent-sample *t*-test to assess alterations of the total topological parameter variation for each topological parameter over a wide range of connection densities. Continually, the *value of p* of <0.05 (uncorrected) was considered statistically significant. Finally, correlation analysis was performed on the clinical scale (FMA-UE) and network attribute indicators in the control group and the BCI group with a significance level of the *p*-value of < 0.05.

## 3. Results

### 3.1. Demographic and clinical characteristics

A total of 40 patients with post-stroke upper extremity hemiparalysis were identified and analyzed in this study. There were no adverse events during the intervention. The patients in the BCI group (five female patients; age: 58.30 ± 11.23 years) and the control group (four female patients; age: 50.90 ± 12.64 years) were included in the statistical analysis. Information on demographic and neurological characteristics is shown in Table 1. No significant differences were observed between the BCI and the control group for variables that included age (*p* = 0.058), sex (*p* = 1.000), duration from stroke onset (*p* = 0.589), etiology (*p* = 0.096), lesion laterality (*p* = 1.000), baseline motor function and ability to participate in activities of daily living (*p* = 0.099), and motor imagination ability (VIQ, *p* = 0.844; KIQ, *p* = 0.956). Both BCI (mean difference, 9.1; *p* = 0.001) and the control group (mean difference, 1.65; *p* < 0.001) showed significant improvement within the group in the Fugl–Meyer Assessment of Upper Extremity scores. After the intervention, patients in the BCI group showed higher scores of the FMA-UE than the control group (*p* = 0.0035; Table 2).

**Table 2:** Statistical description of FMA-UE changes ( $\bar{X}\;$ ± *S*, score).

Group	Sample	Pre-intervention	Post-intervention	*F*	*p*
Control group	20	20.75 ± 10.77	22.40 ± 10.58	16.399	0.001*
BCI group	20	20.80 ± 11.21	29.90 ± 11.08	22.860	<0.001*
*F*	–	0	4.794	–	–
*P*	–	0.989	0.035*	–	–

*p < 0.05.

### 3.2. Alterations In subnetwork organization of The functional connectome

**Figure 4:**
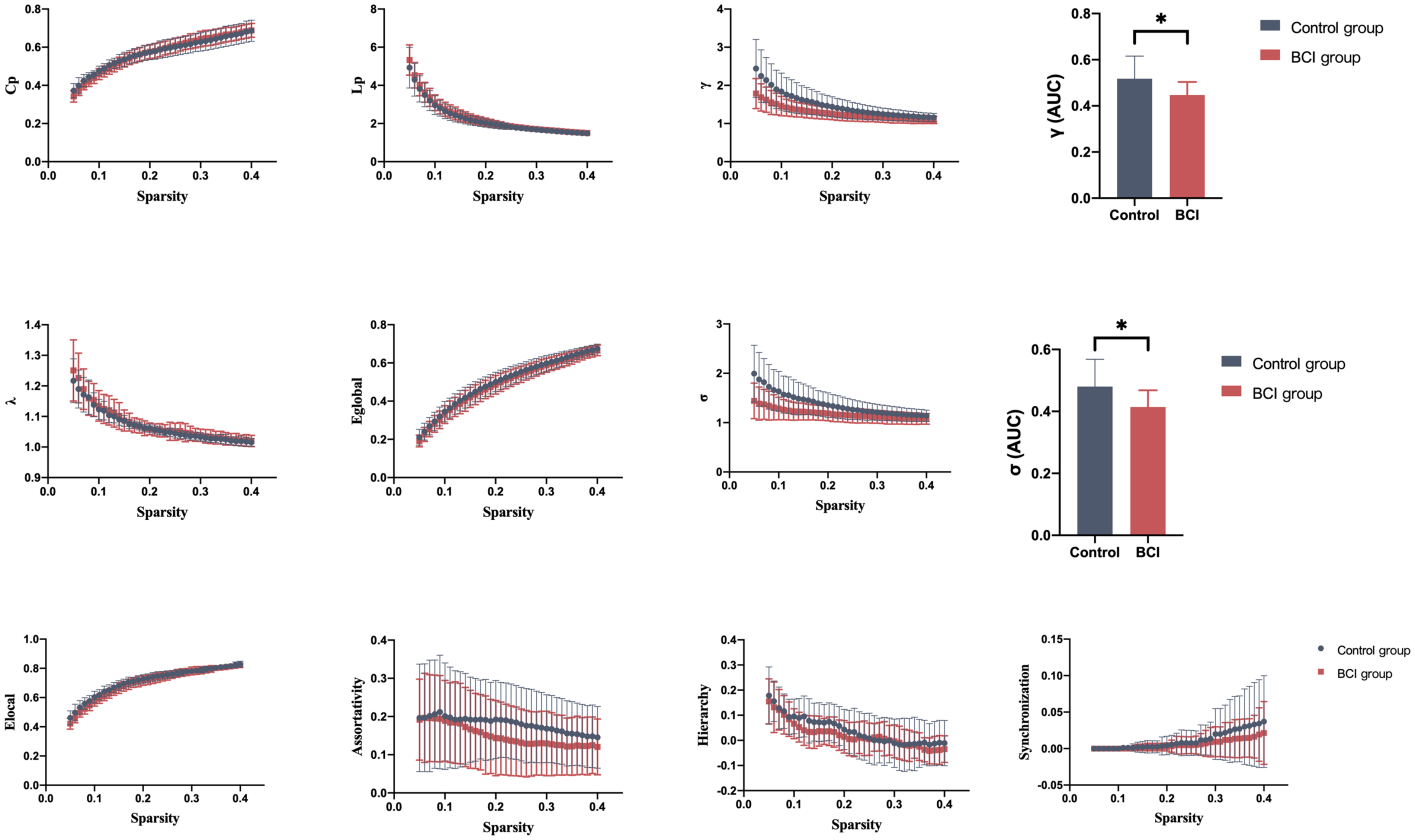
Properties of the functional brain network of the VN with a bin-width sparsity of 0.01. The area under the curve (AUC) displayed no significant differences in Cp, Lp, γ, λ, Eglobal, Elocal, assortativity, hierarchy, or synchronization between the groups. BCI group: red symbols and lines; control group: gray symbols and lines.

**Figure 5:**
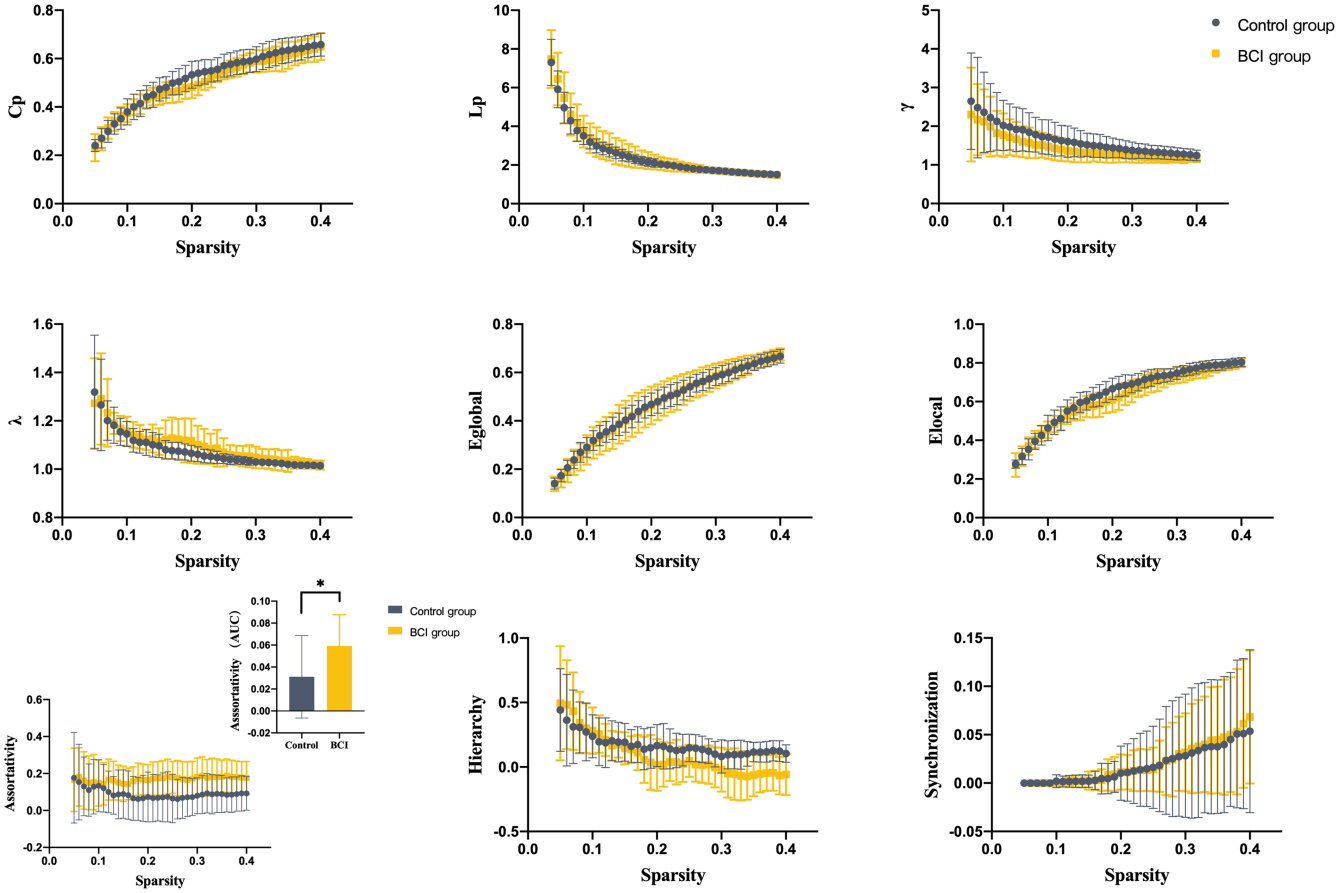
Properties of the functional brain network of the DAN with a bin-width sparsity of 0.01. The area under the curve (AUC) displayed no significant differences in Cp, Lp, γ, λ, Eglobal, Elocal, assortativity, hierarchy, or synchronization between the groups. BCI group: yellow symbols and lines; control group: gray symbols and lines.

We identified seven subnetworks from the resting-state data after the ICA procedure (see Figure 3): the auditory network (AUN), default mode network (DMN), dorsal attention network (DAN), ventral attention network (VAN), frontoparietal network (FPN), sensorimotor network (SMN), and visual network (VN). A comparison of the subnetwork of VN between groups revealed that *γ* (*p* = 0.035) and *σ* (*p* = 0.031) within the VN progressively decreased in the BCI group (see Figure 4). For component DAN, significant differences were found in assortativity (*p* = 0.045) between the groups (see Figure 5). Comparisons of other components found a non-significant difference between the groups.

### 3.3. Correlation analysis

**Figure 6:**
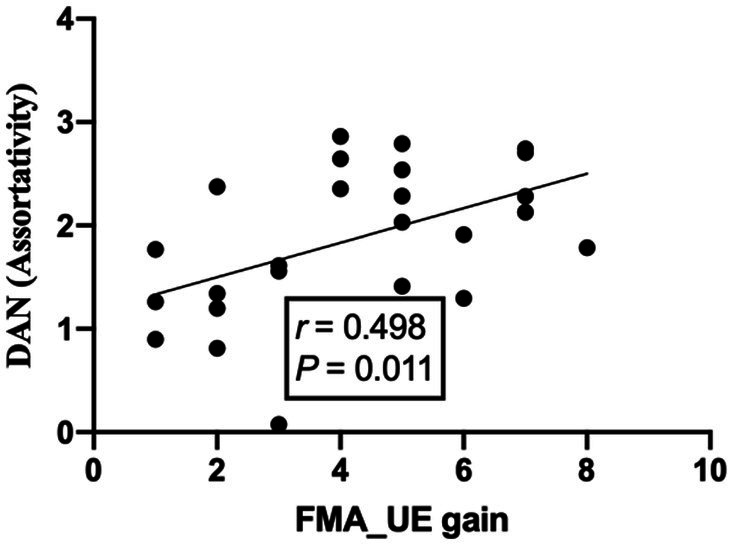
Correlation analysis between DAN network attributes and FMA-UE gains.

The assortativity of the dorsal attention network was positively correlated with the gain of the FMA-UE after treatment, the correlation coefficient was 0.498, and the *p*-value was less than 0.05, which was considered statistically significant (see Figure 6).

## 4. Discussion

This study demonstrates the results of a clinical study investigating the efficacy of BCI compared to conventional therapy for upper extremity stroke rehabilitation. In terms of FMA-UE clinical scale scores, improvements in upper extremity motor function were found in both groups after 10 interventions. The motor function improvement in the BCI group was significantly greater than in the control group. This result is consistent with previous studies on the effectiveness of BCI interventions for upper limb motor function recovery in stroke patients (53). However, Li et al. (54) hold that apparent effects of the BCI intervention can occur at follow-up weeks for post-stroke patients. Add-on therapy of BCI training with conventional therapy may enhance upper extremity motor and brain function recovery in post-stroke patients (55).

After focal brain injury in stroke patients, not only the lesion is affected but also the structure and functional anatomy will be remodeled and reorganized to compensate for the lesion itself and the long-distance effects. New advances in the analysis of functional neuroimaging data allow us to assess *in vivo* the specific contribution of individual brain regions to functional recovery and the effect of treatment on cortical reorganization. Connectivity analysis and network topology studies are important for investigating the effect of stroke on brain networks and helping us understand why some patients recover better than others. Therefore, our study uses graph theory to clarify the impact of BCI on the pathological network configuration of stroke patients, so as to optimize the BCI strategy. It led us to switch attention from motor-related networks to attentional networks, thereby opening the way for the patient’s response to intervention (56).

In the present study, a significant difference in VN and DAN was found after 10-time BCI interventions. Neuroimaging studies have shown that components of multisensory input involve multiple cortical regions that interact with the world through the integration of information from multiple senses (57). Moreover, there was a positive trend between the motor function of FMA-UE and the assortativity of DAN. However, the correlation coefficient of 0.498 is a low correlation. This positive and weak trend is consistent with the study cited in (58), which showed that activation of the dorsal attentional network, increasing patient cognitive engagement and thereby activating the injured cortex at the network level, could facilitate motor-related tasks in subacute stroke patients (59).

The dorsal attention network (DAN) is also called the visuospatial attention network (visuospatial attention network), and the main brain areas include bilateral parietal internal sulcus, central anterior sulcus, and suprafrontal sulcus (frontal eye Active area), which are mainly responsible for providing the top-down attention orientation, participating in exogenous tasks, and continuing activities when prompting clues about when and where to react. The understanding of upper limb and hand movements is mostly considered to consist of two integrated movements, namely reaching and grasping. Each movement is mediated by different neural pathways from the visual to the motor cortex. Milner and Goodale (60) proposed the coexistence of the ventral circuit for object recognition and the dorsal circuit from the visual cortex through the posterior parietal lobe to the premotor and motor areas to visually guide the actions of the object. The latest view based on macaque models and human neuroimaging studies believes that two specialized dorsal parietal and frontal circuits control perception, both of which include the projection of MI. In humans, the dorsolateral circuit connects the anterior intraparietal sulcus (aIPS) and the inferior parietal lobules (IPL) to the ventral anterior motor cortex (PMv) to produce purposeful hand movements.

Neuronal reorganization may occur in brain regions including the ipsilesional and contralesional hemispheres or networks even between network activation during recovery to regain motor function. Therefore, neural circuit modulation is often discussed (61). Meanwhile, this network pattern provides a possible method to control the BCI system for stroke rehabilitation through DAN-based information fusion (58). Therefore, regulation of affected brain regions based on brain networks or connections between networks may play an important role in motor recovery following stroke (62). There is an opinion that modulation of the loop may promote the reorganization of the damaged hemisphere, which may have a positive effect on recovery. Based on this perspective, a restoration model was proposed that links the integrity of the loop pathway and functional restoration to structural reverse.

## 5. Conclusion

Several highlights distinguish this study from former studies. First, we revealed the clinical efficacy of an MI-based BCI system for post-stroke rehabilitation. The BCI system presented patients with a more vivid training experience through auditory cues, motion observations, and multisensory (robotic, auditory, and visual) feedback, allowing subjects to deeply engage in training. Second, we speculated that DAN network participation and the positive correlation between combined inhibition and clinical scores are the ‘priming state’ of motor recovery in patients. Third, we provided a potential BCI optimization procedure (training attention and integration of visual stimulation integration) or neuromodulation stimulation of DAN/VN, which may contribute to a full picture of the key goals of the prescription.

## 6. Limitations

Some limitations of our study merit further discussion. First, for the experimental part of the research, the control group should be treated with an additional equal amount of treatment with reference to the randomized controlled trial to improve the reliability of the experimental results. Second, this study cannot provide real-time EEG signals and offline EEG data to better clarify the treatment effect and brain plasticity of MI-BCI. Third, the number of training sessions was small, yet there was generally no significant change in treatment for 2 weeks. However, we were unable to increase the number of sessions and take a follow-up study due to the limited hospital stay of participants. Furthermore, the device for the rehabilitation of the hand is only used to drive wrist and finger dorsiflexion. They can also be placed in other muscle groups to aid in the motor rehabilitation of other body parts (e.g., elbows and shoulders). Their rehabilitation effect is still unknown, pending further research. Last but not least, the small sample of subjects and a wide range of post-stroke delays, stroke types, and lesion locations limited the further investigation of efficacy.

## Data availability statement

The raw data supporting the conclusions of this article will be made available by the authors, without undue reservation.

## Ethics statement

The studies involving human participants were reviewed and approved by the Medical Ethics Committee of Yueyang Hospital. The patients/participants provided their written informed consent to participate in this study. Written informed consent was obtained from the individual(s) for the publication of any potentially identifiable images or data included in this article.

## Author contributions

J-GX, X-YH, M-XZ, and J-JW designed the study. Z-ZM, X-XX, JM, and C-LS performed the data collection and the data analysis. Z-ZM and J-JW wrote the manuscript for publication. All authors contributed to the article and approved the submitted version.

## Funding

This study was supported by the National Key R&D Program of China (Grant nos: 2018YFC2001600 and 2018YFC2001604), the National Natural Science Foundation of China (Grant nos: 81802249, 81871836, 81902301, and 82172554), the Shanghai Science and Technology Committee (Grant no: 22010504200), the Shanghai Rising-Star Program (Grant no: 19QA1409000), the Shanghai Municipal Commission of Health and Family Planning (Grant nos: 2018YQ02 and 201840224), and the Program of Shanghai Academic Research Leader (Grant no: 19XD1403600).

## Conflict of interest

The authors declare that the research was conducted in the absence of any commercial or financial relationships that could be construed as a potential conflict of interest.

## Publisher’s note

All claims expressed in this article are solely those of the authors and do not necessarily represent those of their affiliated organizations, or those of the publisher, the editors and the reviewers. Any product that may be evaluated in this article, or claim that may be made by its manufacturer, is not guaranteed or endorsed by the publisher.

## Abbreviations

BCI: brain computer interface
FMA: Fugl–Meyer Assessment
FMA-UE: Fugl–Meyer Assessment of Upper Extremity
FMA-LE: Fugl–Meyer Assessment of Lower Extremity
ICA: independent component analysis
MI: motor imagery
SMRs: sensorimotor rhythms
ERD: event-related desynchronization
NF: neurofeedback
EEG: electroencephalography
MEPs: motor evoked potentials
NIHSS: National Institute of Health stroke scale
MMSE: mini-mental state examination
MBI: modified Barthel Index
rs-FMRI: resting-state functional magnetic resonance imaging
TR: repetition time
FOV: field of view
FA: flip angle
GRETNA: GRaph thEoreTical Network Analysis
MNI: Montreal Neurological Institute
ICs: independent components
PCA: principal component analysis
RSNs: resting-state networks
AUN: auditory network
DMN: default mode network
DAN: dorsal attention network
VAN: ventral attention network
FPN: frontoparietal network
SMN: sensorimotor network
VN: visual network
AUC: area under the curve
aIPS: anterior intraparietal sulcus
IPL: inferior parietal lobules
PMv: ventral anterior motor cortex
